# Ureterorenoscopic (URS) Lithotripsy and Balloon Dilation Cause Acute Kidney Injury and Distal Renal Tubule Damage: A Prospective Study

**DOI:** 10.1155/2022/5505969

**Published:** 2022-09-14

**Authors:** Ho-Shiang Huang, Ze-Hong Lu, Chan-Jung Liu

**Affiliations:** Department of Urology, National Cheng Kung University Hospital, College of Medicine, National Cheng Kung University, Tainan, Taiwan

## Abstract

Ureterorenoscopy (URS) is believed to be a safe and effective procedure for treating ureteral stones or ureteral strictures. Rapidly increasing intrarenal pressure during URS may have a negative impact on the kidney, but its effect on renal function is not well known. The aim of this study was to evaluate whether URS balloon dilation or lithotripsy could cause acute kidney injury (AKI), which was evaluated using urine neutrophil gelatinase-associated lipocalin (NGAL), and renal tubular damage, which was evaluated using urine *α*-glutathione S-transferase (GST) and *π*GST. This prospective study included 207 patients with a mean age of 53.8 years between September 2012 and June 2013. Four groups were included: the ureteral stricture group (group 1), the ureteral stone group (group 2), and two control groups. URS increased urine NGAL (uNGAL) levels on days 1 and 14 in both groups, and only elevated uGST levels were noted on day 14 after URS lithotripsy (URS). On day 14, the difference between low-grade and high-grade hydronephrosis was significant in group 1 (*p* < 0.001) compared to that in group 2 (*p* = 0.150). Multivariate logistic regression analysis revealed that age, baseline estimated glomerular filtration rate (eGFR), and stone size > 1.0 cm were associated with the complete recovery of hydronephrosis after URS on day 14. Patients with ureteral stones with preserved renal function had more AKI than those with impaired renal function. However, there was no significant difference in URS-related AKI between the ≤1 cm and >1 cm subgroups. In addition, urine *α*GST and *π*GST levels were both significantly higher in the stone > 1 cm subgroup than in the ≤1 cm subgroup. In conclusion, URS laser lithotripsy and balloon dilatation resulted in AKI and renal tubular damage on day 14, although post-URS double-J (DBJ) stenting was performed in every patient.

## 1. Introduction

Impairment of urinary flow due to urinary tract obstruction, referred to as obstructive uropathy, is a manifestation of various kidney and ureteral diseases [1]. The progression of renal dysfunction after relief from obstructive uropathy has been widely studied. When experimental animals undergo 24 hours of unilateral ureteral obstruction, a decline in renal hemodynamic and tubular functions is observed [1, 2]. The glomerular filtration rate (GFR) is directly affected by intrapelvic pressure and decreases to zero as pressure progressively increases, whereas renal blood flow does not respond directly to intrapelvic pressure [3]. Furthermore, calcium oxalate (CaOx) stone disease per se can induce renal tubular damage and renal interstitial fibrosis, which has been found in both stone patients and experimental animals [4–6]. Therefore, profound kidney damage is more likely to occur in patients with obstructive uropathy caused by CaOx ureteral stones.

Ureterorenoscopic lithotripsy (URSL) is a safe, effective, and minimally invasive method for the treatment of ureteral stones [7]. Good irrigation is vital for ureteral dilatation and instrument passage, and irrigation is required to provide clear vision [8]. However, the application of high-pressure irrigation during URS can cause an accumulation of renal intrapelvic fluid and increase intrapelvic pressure significantly [8]. High-pressure irrigation during URS can cause irreversible damage to the urothelium and renal parenchyma [9, 10]. Ureteral stricture is another major cause of obstructive uropathy [11]. Similar to ureteral stones, ureteral strictures are associated with kidney injury and fibrosis [12]. Various management strategies for ureteral strictures can be used based on the preference and experience of urologists. Balloon dilation of the ureter is a well-accepted surgical technique for resolving ureteral stricture [13]. However, many urologists prefer internal stents, such as double-J stents (DBJ), in most circumstances to treat ureteral strictures rather than balloon dilation because of the potential risk of ureteral injury. Taken together, although both ureteral stones and ureteral strictures are associated with kidney injury, it is still controversial whether their treatments, URSL and balloon dilation, attenuate or aggravate the kidney injury. Whether URSL or balloon dilation of the ureteral stricture causes more profound AKI and renal tubular damage has not yet been fully investigated.

Acute kidney injury (AKI) is an important clinical issue associated with short- and long-term morbidity and mortality [14]. Neutrophil gelatinase-associated lipocalin (NGAL) has emerged as the most promising biomarker of AKI [15]. Growing evidence suggests that urinary NGAL is an early and accurate biomarker for predicting AKI [15–17]. Alpha-glutathione S-transferase (*α*GST) is a cytosolic enzyme that has proven to be a useful marker of chemically induced tubular damage, particularly in the S3 segment of the proximal tubule [18]. Pi-GST (*π*GST) is also a cytosolic enzyme that is mainly localized in the distal tubules and collecting ducts, and its presence in the proximal tubules is limited [19]. In this prospective study, we investigated whether URS or URS balloon dilation would cause AKI (evaluated by urine NGAL levels) and renal tubular damage (evaluated by urine *α*GST and *π*GST levels). We also evaluated the variables that contributed to the complete recovery of hydronephrosis after URS and the impact of hydronephrosis. Therefore, the present study is aimed at providing a comprehensive understanding of the impact of URS procedures on the renal function.

## 2. Patients and Methods

This was a prospective case-control study, and the protocol of this study was approved by the Institutional Ethics Review Board of the National Taiwan University Hospital (Registry Number: 201205117RIC), and all the participants were provided with a written informed consent.

### 2.1. Study Design and Populations

We prospectively enrolled patients from a single tertiary medical center between September 2012 and June 2013. All patients were admitted for URS holmium: yttrium-aluminum-garnet (Ho: YAG) laser lithotripsy or balloon dilation due to acute unilateral hydronephrosis caused by ureteral stones or strictures, which was confirmed by renal ultrasonography, intravenous urography (IVU), or noncontrast computed tomography (CT). The exclusion criteria were as follows: (1) a history of urolithiasis; (2) acute pyelonephritis or an associated urinary tract infection; (3) nephrostomy tube insertion before URSL or current indwelling; (4) hydronephrosis caused by infravesical obstruction, uterine myoma, malignancy, or other retroperitoneal etiology; (5) stone analysis showing no CaOx; and (6) other inflammatory or malignant diseases.

### 2.2. Hydronephrosis Classification

The grade of hydronephrosis was classified according to McIlroy et al. [20]: grade 1, enlargement of the calices with preservation of the renal papillae; grade 2, rounding of the calices with obliteration of the renal papillae; and grade 3, caliceal ballooning with cortical thinning. All renal sonographic examinations and determination of the hydronephrosis grade were performed by a single urologist. Two weeks after surgery and DBJ removal, post-URS sonography was performed.

### 2.3. Patient Grouping

**Figure 1:**
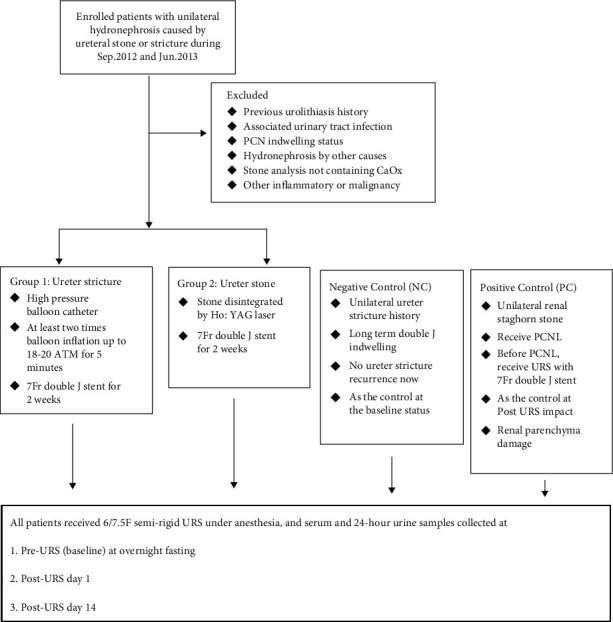
Consort flow diagram.

In the current study, all subjects were categorized into four groups: two study groups (the ureter stricture group and the ureter stone group) and two control groups (positive and negative) (Figure 1). The diagnosis of obstructive uropathy was established *via* IVU or noncontrast CT scan two to three weeks after the first outpatient department interview. One week after the IVU or CT scan, the patients returned to our outpatient department to confirm that obstructive uropathy was associated with ureteral stones or strictures. All patients were enrolled in the ureteral stricture group (group 1) or ureteral stone group (group 2) based on the image results.

Serum and 24-hour urine samples were collected at three time periods. Pre-URS (baseline) samples were collected after overnight fasting, one-day post-URS (day 1) samples, and two-week post-URS (day 14) samples were collected from all patients while they followed a normal diet. There was at least a seven to ten-day interval between the IVU examination and the subsequent collection of blood and urine samples. Maximal stone length was assessed based on IVU or abdominal CT images.

In the current study, we used two groups of controls: a negative control (NC) and a positive control (PC). The NC, serving as the control at the baseline status, were those with a history of unilateral ureteral stricture with long-term unilateral DBJ catheter indwelling. A total of 14 patients were replaced with 7-Fr DBJ catheters under anesthesia, and no recurrence of ureteral stricture was simultaneously confirmed by URS and retrograde pyeloureterography at the same time. We used patients with unilateral renal staghorn stones who underwent percutaneous nephrolithotomy (PCNL) as the PC, and they served as the controls mainly during the post-URS period to investigate the impact of URS on the changes in these biomarkers. Before PCNL, the PC underwent URS with a 7-Fr ureteral DBJ indwelling. The NC group included patients without any evidence of obstructive uropathy. Otherwise, PC represented those who experienced the most severe renal injury because of inevitable renal volume damage during PCNL.

### 2.4. The Procedure for URS

URS was performed on all enrolled patients. After anesthesia, a 6F/7.5F semirigid URS (Richard Wolf Medical Instruments Corporation, Vernon Hills, IL, USA) was introduced into the ureter retrogradely along with a safety guide wire to locate the ureteral stone or site of the ureteral stricture. Normal saline irrigation through the URS was performed using a hand-held syringe to enhance instrument passage and maintain clear vision.

In stone patients (group 2), the ureteral stone was disintegrated by the application of a Ho:YAG laser (Odyssey, Convergent Laser Technologies, Alameda, CA, USA) through URS. After the ureteral stone was disintegrated, ureteral patency was examined using URS to ensure that there was no ureteral injury caused by laser lithotripsy.

In patients with ureteral stricture (group 1), a high-pressure balloon catheter (UroMAx Ultra; Boston Scientific, Natick, MA, USA) was used to relieve the stricture. Balloon dilation was applied at least twice with balloon inflation pressure up to 18–20 atm for five minutes each time under fluoroscopy to ensure that all segments of the ureteral stricture were relieved.

A urologist performed all endoscopic interventions. Each patient had a 7-Fr ureteral catheter placement (Cook Medical, Bloomington, Indiana, USA), which was left for two weeks.

### 2.5. Biochemical Analysis and Renal Function Determination

Every enrolled patient's serum creatinine (Cr) levels were measured twice, at baseline and two weeks after URS, and the eGFR was calculated using the following formula:

186 × (serum Cr)^−1.154^ × (age)^−0.203^ × (0.742 if female) × (1.210 if African − American) [2, 3].

Commercial kits were used to determine the urine levels of NGAL (NGAL ELISA Kit, BioPorto Diagnostics A/S, Copenhagen, Denmark) and the urine levels of stone-induced renal tubular damage markers, namely, urinary *α*GST (Alpha GST EIA, Argutus Medical, Dublin, Ireland), which is a marker of proximal tubular damage, and *π*GST (Pi GST EIA, Argutus Medical, Dublin, Ireland), which is a marker for distal tubular damage. Urinary *α*GST and *π*GST were examined at baseline and two-week post-URS (day 14), and all assays were performed in duplicate. Urine NGAL (uNGAL) levels were examined at baseline and post-URS days 1 and 14.

### 2.6. Statistical Analysis

Continuous variables are presented as mean values ± standard deviation, whereas categorical variables are presented as frequencies. Two-sample comparisons between patients with kidney stones and controls were performed using the unpaired Student's *t*-test, Mann-Whitney *U*-test, or Fisher's exact probability test, as appropriate. Comparisons across the three groups were performed using the chi*-*square test for categorical variables and analysis of variance (ANOVA) or Kruskal–Wallis test for continuous variables, depending on the distribution of the variable. Logistic regression was used for univariate and multivariate analyses to identify the factors affecting hydronephrosis recovery (hydronephrosis degree = 0). Pearson's correlation coefficient (*γ*) was used to assess the correlation between the clinical variables. In all tests, *p* value < 0.05 was considered statistically significant.

## 3. Results

A total of 220 patients were enrolled in the current study, mainly male patients (67.3%), with a mean age of 53.80 years (Table 1). The male-to-female ratio in each group was 1.17 (group 1), 2.53 (group 2), 3.67 (PC), and 1.6 (NC). There were no significant differences in age, eGFR, body mass index, or 24-hour urine output between the four groups. In group 1, the number of cases of ureteral stricture was 31, 3, and 18 in the upper, middle, and lower ureter, respectively, whereas the number of ureteral stone locations in group 2 was 79, 42, and 20, respectively. The mean maximum stone length was 0.9 cm.

### 3.1. The Impact of Different Degrees of Hydronephrosis on Kidney Injury Biomarkers

Table 2 shows the comparison of different kidney injury biomarkers in groups 1 and 2 with different degrees of hydronephrosis. Compared to the NC group, significantly elevated uNGAL levels were noted in patients with stones (group 2) with moderate-to-severe hydronephrosis and severe hydronephrosis was noted in the patients with ureter strictures (group 1). A significant elevation of renal tubular damage markers (both u*α*GST and u*π*GST) was also found in patients with stones along with moderate-to-severe hydronephrosis, but we did not find any difference in stricture patients. Interestingly, when compared with patients with strictures, u*α*GST and u*π*GST levels were significantly higher in patients with stones along with moderate-to-severe hydronephrosis, whereas there was no significant difference in uNGAL levels between the groups.

### 3.2. Changes in Urine NGAL, *α*GST, and *π*GST Levels at Different Time Periods

**Figure 2:**
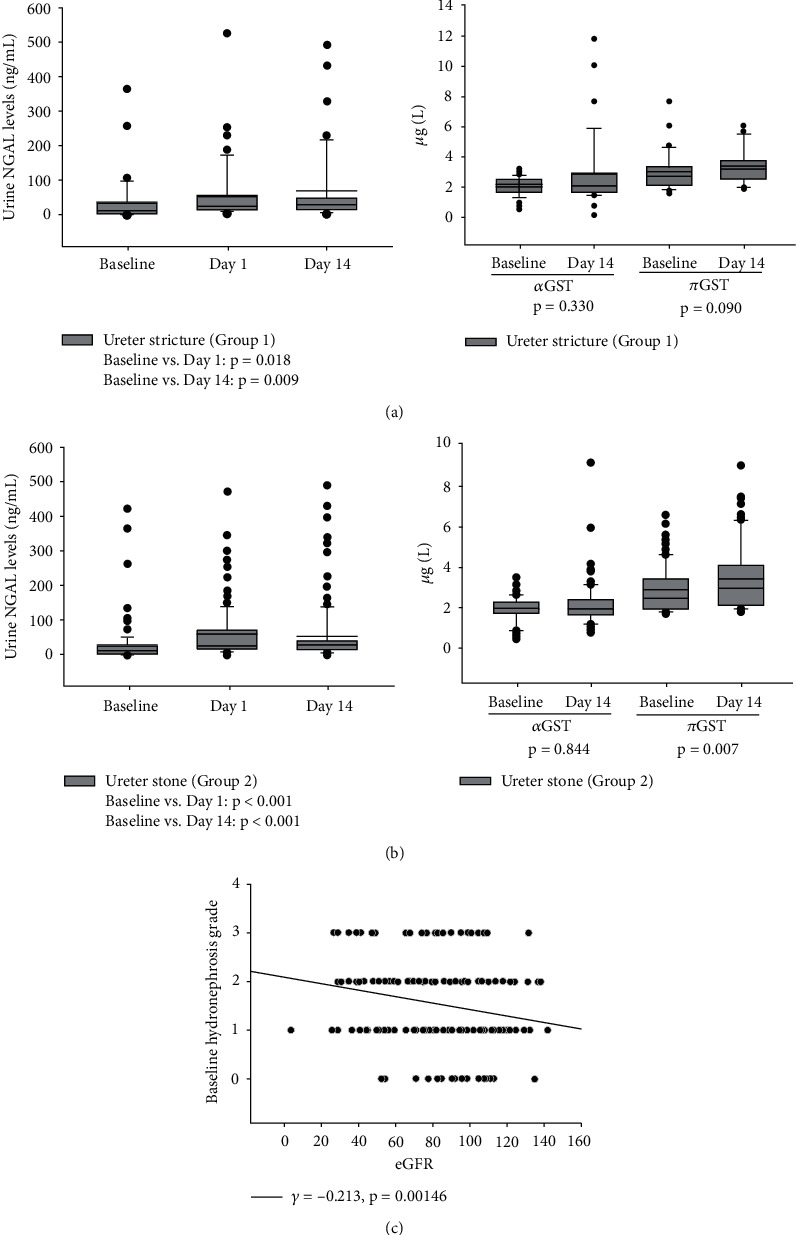
(a, b) Changes in urine NGAL, *α*GST, and *π*GST levels at baseline, day 1, and day 14 after URS in both groups; (c) correlation between baseline hydronephrosis grade and baseline eGFR in all cohorts.

In the ureter stricture group, uNGAL increased significantly on days 1 (*p* = 0.018) and 14 (*p* = 0.009) when compared to the baseline, but there was no significant difference between days 1 and 14 (Figure 2(a)). However, we did not find any significant increase in u*α*GST or u*π*GST on day 14 compared to baseline.

In the ureter stone group, we also found a similar result as in the stricture group, which showed significantly elevated uNGAL on day 1 (*p* < 0.001) and day 14 (*p* < 0.001) when compared to baseline (Figure 2(b)). Most importantly, in groups 1 and 2, there was no significant decrease in uNGAL levels from day 1 to 14, indicating that kidney injury related to URS surgery persisted for at least two weeks. In contrast, u*π*GST levels increased significantly on day 14 (*p* = 0.007) in the stricture group, but this increase was not observed in u*α*GST.

Larger stones may cause more severe kidney obstruction and kidney injury. Hence, we investigated the impact of stone size on changes in urinary biomarkers (Supplementary Figures 1A and 1B). The change in uNGAL level at different time periods was nearly the same in both ureteral stone ≤ 1 cm and ureteral stone > 1 cm. However, only in the ureteral stone ≤ 1 cm subgroup that the level of u*π*GST was significantly elevated on day 14 compared with the baseline, and no difference between the baseline and day 14 was found in u*α*GST. We further compared the three urinary biomarkers between the ureteral stone ≤ 1 cm and ureteral stone > 1 cm subgroups. The baseline levels of uNGAL and u*α*GST revealed significant differences between the ≤1 cm and >1 cm subgroups, and the level was significantly higher in the larger stone size group (Supplementary Figure 1C). There was no significant difference in the baseline level of u*π*GST; however, there was a nonsignificant trend (*p* = 0.085) (Supplementary Figure 1C).

Using Pearson correlation analysis, we found that baseline eGFR and hydronephrosis grade had a significant inverse correlation (*γ* = −0.213, *p* = 0.0015) (Figure 2(c)).

### 3.3. Predictors of Complete Recovery of Hydronephrosis after URS

We conducted univariate and multivariate logistic regression analyses to identify the predictors of complete recovery from hydronephrosis after URS (Supplementary Table 1). We found that only three factors, including age (odds ratio (OR) = 0.96, *p* = 0.002), baseline eGFR (OR = 1.04, *p* < 0.001), and stone size > 1.0 cm (OR = 2.56, *p* = 0.024), had a statistically significant effect on the complete recovery of hydronephrosis after URS and DBJ removal.

**Figure 3:**
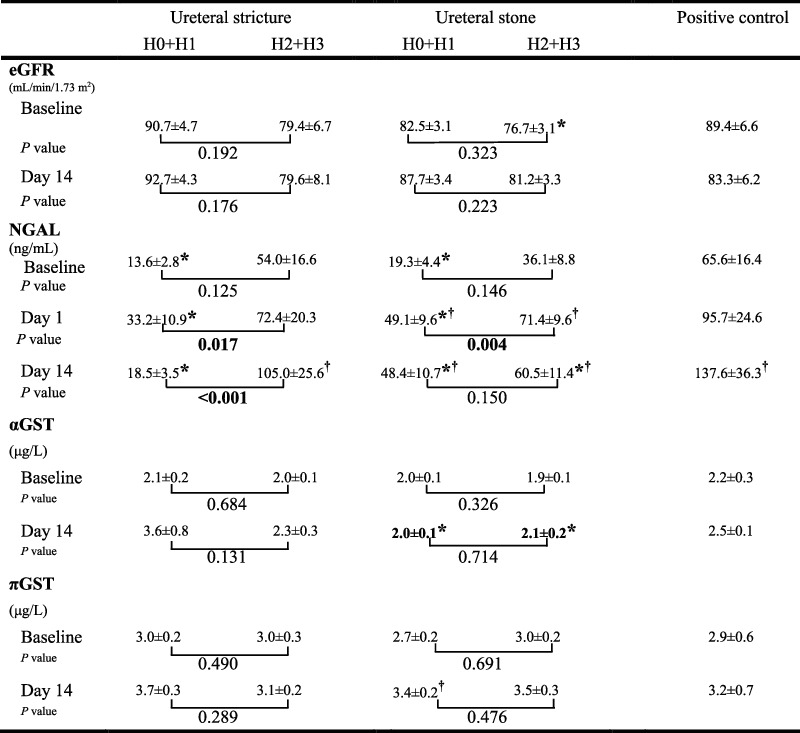
Impact of hydronephrosis degree on eGFR, AKI marker, and urinary kidney damage markers in each group.

We also investigated the effects of different degrees of hydronephrosis on kidney injury and renal function (Figure 3). Each group was stratified into H0+H1 and H2+H3 subgroups according to the baseline degree of hydronephrosis. There was no significant difference in eGFR between the two subgroups at baseline and on day 14 in each group; however, the H2+H3 subgroup of the stone group had a lower eGFR than the PCs. The baseline uNGAL levels of the H0+H1 subgroup in groups 1 and 2 were all significantly lower than those of the PC, but those of the H2+H3 subgroup were not significantly different from the PCs. Compared to the two subgroups in each group, the uNGAL level in group 1 remained significantly higher in the H2+H3 subgroup on days 1 and 14, whereas in group 2, the level of uNGAL was significantly higher in the H2+H3 subgroup only on day 1. There was no significant difference in the u*α*GST and u*π*GST levels between the two subgroups of groups 1 and 2, although group 2 had lower u*α*GST levels on day 14 in both subgroups.

### 3.4. The Impact of Baseline eGFR on Kidney Injury

We evaluated the changes in different urine biomarkers (*Δ*) in the baseline eGFR ≥ 60 and eGFR < 60 subgroups in both groups (Supplementary Table 2). The changes were defined as the level on day 14 minus the baseline level. Only the *Δ*NGAL level in the ureter stone group was significantly higher in the eGFR ≥ 60 subgroup than in the eGFR < 60 subgroup. The levels of *Δα*GST and *Δπ*GST were not significantly different between the two subgroups in either study group.

## 4. Discussion

In the present study, we found that patients with ureteral stones or strictures experienced AKI after URS, which was indicated by elevated uNGAL levels. The impact of URS persisted for at least 14 days and had higher impact on patients with moderate-to-severe hydronephrosis. To clarify a possible explanation for these significant findings, we first determined the characteristics of NGAL [21]. NGAL belongs to the lipocalin superfamily and is secreted by the kidneys, gastrointestinal tract, and respiratory tract. NGAL is rapidly filtered by glomeruli, reabsorbed efficiently by proximal tubules, and finally secreted with only 0.1–0.2% into the urine in the thick ascending limb of the loop of the Henle and collecting ducts under normal conditions. In response to AKI, urine NGAL levels rapidly increased 15 to nearly 100-fold within two hours and serum NGAL increased nearly 30-fold within two hours [22, 23]. The time required for uNGAL reduction varies among different studies, ranging from 24 hours to 14 days [20, 24]. In most studies, uNGAL levels started to decrease 24 hours after treatment. However, in the present study, one of the most significant findings was that an increase in uNGAL persisted for 14 days in both the ureteral stone and ureteral stricture groups. Previous studies have suggested that the ability of uNGAL to provide accurate identification of AKI depends on normal baseline renal function, and participants in both the ureteral stone and ureteral stricture groups had normal baseline renal function. Consequently, the persistent increase in uNGAL in both groups was suggestive of silent and prolonged kidney injury after URS surgery. Although adjusting uNGAL values for serum NGAL may have been instructive in this regard, serum NGAL was not measured in the present study; several authors have mentioned that extrarenal sources of NGAL have generally been considered to have limited significance to urinary NGAL levels. A possible explanation for this previously unreported result may be related to intrarenal pelvic pressure (IPP). Irrigation during URS increases IPP, possibly leading to intrarenal, pyelovenous, and pyelolymphatic backflow as well as kidney injury [9]. Previous studies have found that elevated IPP during URS causes irreversible and harmful effects on the kidney, and even moving the URS in the ureter without irrigation could increase IPP by 20–25 mmHg [25]. Our results are compatible with those of previous studies, in which the URS procedure induced kidney injury and the elevation in uNGAL can be found as early as one hour after URS surgery [26]. However, we observed that the deleterious effects of URS lasted for 14 days, and the underlying mechanism remains uncertain. Larger studies are needed to address this question.

For decades, numerous biomarkers, except Cr, have been used to evaluate kidney function and predict AKI prognosis. When the cell wall integrity of the distal and proximal tubules is damaged, the constitutive cytoplasmic enzymes, *π*- and *α*GST, are enriched in renal tubular epithelial cells and detected in the urine [27]. As highlighted in our previous study, elevated u*α*GST was noted in kidney stone patients, but not in u*π*GST, compared with controls [28]. Previous studies have evaluated the predictive ability of urinary biomarkers, including *α*GST and *π*GST, in children with congenital unilateral hydronephrosis secondary to ureteropelvic junction obstruction [29]. The results revealed that u*α*GST was significantly increased in all patients with any degree of hydronephrosis compared to controls. u*π*GST was increased only in patients with moderate-to-severe hydronephrosis. In the present study, urinary *α*GST and *π*GST were significantly higher in patients with larger renal stones than in those with smaller stones. However, u*π*GST, but not u*α*GST, increased until 14 days after URSL surgery. Possible explanations include distal renal tubule injury caused by a CaOx renal stone [30]. Previous studies have revealed that CaOx dihydrate (COD) crystals can adhere to the apical surface of distal renal tubular epithelial cells, which can lead to crystal growth and aggregation, resulting in kidney stone formation [31]. Further studies are needed to confirm these unanswered questions.

URS is defined as the endoscopic visualization of the ureter and renal pelvis for diagnostic and therapeutic purposes [32]. URS is thought to be a safe and effective surgery for the treatment of ureteral stones. However, the optimal duration and indication for post-URS stenting are controversial. Some authors have suggested that post-URS DBJ stents are only used in patients who are at an increased risk of complications, and most urologists may favor their use for one to two weeks after URS [32, 33]. Our results suggest that even after two weeks of DBJ indwelling, URS-induced AKI persisted in patients with ureteral stones or ureteral strictures. Most noteworthy in the present study was that we used NCs and PCs to assist our interpretation of the results. The NCs represented no obstructive uropathy, and we compared the two study groups with NCs to validate whether these urinary biomarkers increased under these clinical conditions. Our results showed that both ureter stone and stricture patients with a higher degree of hydronephrosis had higher levels of all urinary biomarkers. Furthermore, we used PCNL patients as the PCs because PCNL is known to create a renal tract to assess intrapelvic renal stones, and it certainly causes considerable kidney injury. There are two interesting findings regarding PCs. First, although we did not observe any decrease in the baseline eGFR from PCs, the baseline level of uNGAL had already significantly increased in PCs compared with mild obstructive uropathy patients in both groups. This indicated that the presence of renal stones could lead to progressive renal injury. We used the GFR criteria instead of urine output criteria because urine output is easily affected by postoperative hydration, continuous Foley normal saline irrigation, and urine collection recording errors. Second, in the ureter stricture group with moderate-to-severe hydronephrosis, uNGAL nearly doubled 14 days after URS, similar to the PCs. The level of uNGAL on day 14 in the ureter stricture group with moderate-to-severe hydronephrosis was >100 ng/mL, which was even higher than the level on day 1 in the PCs. This finding was not noted in other patients, whereas uNGAL levels decreased 14 days after a peak on post-URS day 1 in other patients. Our results also imply that URS balloon dilation may cause more AKI than URSL in patients with high-grade hydronephrosis on day 14 (Figure 3). The possible explanations underlying this finding are possibly related to the URS procedures. Patients with ureteral stricture with moderate-to-severe hydronephrosis usually have more strictures and tortuous ureters. Urologists must use more forceful saline irrigation to distend the ureter lumens and facilitate the URS to pass through the stricture site. A sudden increase in irrigation flow causes acute high IPP (>200 cmH_2_O), which causes diffuse flattening of the caliceal urothelium, renal tubule dilation, and renal glomerulus compression [34]. In contrast, urologists need to maintain adequate irrigation and prevent upward migration of ureteral stones during URSL, and IPP can be controlled under 120 cmH_2_O, which does not cause renal injury [25]. In a recent porcine experimental study, under gravity irrigation and manual pumping, the maximal IPP during URS was 30 and 105 cmH_2_O, respectively [8]. Therefore, our findings suggest that the effect of URS on severe ureteral strictures complicated by high-grade hydronephrosis is related to prolonged kidney injury.

Obstructive nephropathy, which often presents as hydronephrosis, refers to an anatomical or functional obstruction of the kidney and leads to progressive kidney injury. Various factors can cause obstructive nephropathy, including ureteral stones, strictures, and tumors. Few studies have investigated the factors associated with the development of ipsilateral postoperative hydronephrosis in patients who underwent URS [35, 36]. The incidence was reported to range from 15.0% to 32.1% [37]. A larger stone size, longer operation time, and prior ipsilateral URS procedure were associated with an increased risk of postoperative hydronephrosis. In our cohort, we also found that a larger stone size increased the risk of postoperative hydronephrosis (OR 2.56, 95% CI 1.13–5.77, *p* = 0.024). Younger age and better baseline eGFR were associated with an increased risk of postoperative hydronephrosis. Although the association was relatively weak, further studies are needed to dissect this particular result.

This study had several limitations. First, we did not detect or record IPP during the operation. The relationship between blood pressure and the grade of renal injury cannot be established. Second, there is no standard irrigation protocol for URS. Irrigation pressure is created by gravity, manual, or pumping devices, and hence, the variation in pressure amplitude is uncertain. Third, we did not observe a significant change in eGFR after URS. However, mild renal injury did not result in differences in serum creatinine levels. Other material clearances, such as inulin, require further study and evaluation.

## 5. Conclusions

Although eGFR did not significantly change after URS surgery, uNGAL persisted at elevated levels for two weeks in both the ureteral stricture and ureteral stone groups, which suggested that URS procedures could cause kidney injury. With respect to the renal tubular damage marker, only the u*π*GST level was elevated on day 14 in the ureteral stone group. Patients with ureteral stones with preserved renal function suffered more uNGAL changes (i.e., *Δ*NGAL) after URS. Taken together, both URSL and URS balloon dilation could lead to kidney injury and renal distal tubule damage for up to two weeks, even though DBJ indwelling persisted in both groups.

## Figures and Tables

**Table 1:** Clinical characteristics of the study population.

	Negative control	Ureteral stricture (group 1)	Ureteral stone (group 2)	Staghorn stone (positive control)
Number (%)	13 (5.9)	52 (23.6)	141 (64.1)	14 (6.4)
Hydronephrosis, *n* (%)				
Grade 0	13 (100)	13 (25.0)	5 (3.5)	0 (0)
Grade 1	0 (0)	17 (32.7)	61 (43.3)	11 (78.6)
Grade 2	0 (0)	13 (25.0)	58 (41.1)	3 (21.4)
Grade 3	0 (0)	9 (17.3)	17 (12.1)	0 (0)
Age, years, mean (SD)	51.7 (2.6)	53.0 (2.0)	54.5 (1.1)	59.1 (2.7)
Gender, *n* (%)				
Male	8 (61.5)	28 (53.8)	101 (71.6)	11 (78.6)
Female	5 (38.5)	24 (46.2)	40 (28.4)	3 (21.4)
eGFR, mL/min/1.73 m^2^, mean (SD)	92.0 (6.6)	87.0 (4.0)	86.9 (3.5)	85.1 (8.7)
BMI, mean (SD)	25.9 (1.2)	24.6 (0.5)	24.3 (2.1)	24.5 (1.0)
Stone size, cm, mean (SD)	-	-	0.9 (0.5)	5.4 (0.7)
Location (stone or stricture), n (%)				
Upper	-	31 (59.6)	79 (56.0)	-
Middle	-	3 (5.8)	42 (29.8)	-
Lower	-	18 (34.6)	20 (14.2)	-

Abbreviations: BMI: body mass index; eGFR: estimated glomerular filtration rate; U/O: urine output.

**(a):** 

Part 1. Compared with negative control (NC)			
NGAL	*p* value		*p* value
Stone H0 vs. NC	0.456		
Stone H1 vs. NC	0.153	Stricture H1 vs. NC	0.113
Stone H2 vs. NC	**0.035**	Stricture H2 vs. NC	0.390
Stone H3 vs. NC	**0.006**	Stricture H3 vs. NC	**0.007**
*α*GST			
Stone H0 vs. NC	0.241		
Stone H1 vs. NC	0.058	Stricture H1 vs. NC	0.385
Stone H2 vs. NC	**0.002**	Stricture H2 vs. NC	0.848
Stone H3 vs. NC	**0.000**	Stricture H3 vs. NC	0.352
*π*GST			
Stone H0 vs. NC	0.332		
Stone H1 vs. NC	0.059	Stricture H1 vs. NC	0.080
Stone H2 vs. NC	**0.007**	Stricture H2 vs. NC	0.604
Stone H3 vs. NC	**0.000**	Stricture H3 vs. NC	0.440

**(b):** 

Part 2. Comparison between ureteral stone (group 2) and ureteral stricture (group 1)	
NGAL	*p* value
Stone H1 vs. stricture H1	0.153
Stone H2 vs. stricture H2	0.219
Stone H3 vs. stricture H3	0.452
*α*GST	
Stone H1 vs. stricture H1	0.064
Stone H2 vs. stricture H2	**0.000**
Stone H3 vs. stricture H3	**0.000**
*π*GST	
Stone H1 vs. stricture H1	0.051
Stone H2 vs. stricture H2	**0.003**
Stone H3 vs. stricture H3	**0.000**

Abbreviations: NC: negative control; H0: grade 0 hydronephrosis; H1: grade 1 hydronephrosis; H2: grade 2 hydronephrosis; H3: grade 3 hydronephrosis.

Bold value represents statistically significant *p* < 0.05.

## Data Availability

Data are available within the article or its supplementary materials.

## Supplementary Materials

Supplementary Materials Supplementary Figure 1: (A, B) impact of ureteral stone size on uNGAL, u-*α*GST, and u-*π*GST levels in the ureteral stone group; (C) comparing the urine biomarkers at different time periods between subgroups of stones≦1 cm and stones > 1 cm in group 2. Supplementary Table 1: univariate and multivariate logistic regression analysis for the predictors of complete recovery of hydronephrosis (hydronephrosis grade = 0 on day 14 after DBJ removal) after URS. Supplementary Table 2: impact of baseline eGFR on changes in urinary renal damage markers in both groups.
