# Supplementary material for: Ureterorenoscopic (URS) Lithotripsy and Balloon Dilation Cause Acute Kidney Injury and Distal Renal Tubule Damage: A Prospective Study
Source: Biomed Res Int. 2022 Sep 14;2022:5505969. doi: 10.1155/2022/5505969 (PMC9492427; doi:10.1155/2022/5505969)
Supplement: Supplementary Materials — Supplementary Figure 1: (A, B) impact of ureteral stone size on uNGAL, u-αGST, and u-πGST levels in the ureteral stone group; (C) comparing the urine biomarkers at different time periods between subgroups of stones≦1 cm and stones > 1 cm in group 2. Supplementary Table 1: univariate and multivariate logistic regression analysis for the predictors of complete recovery of hydronephrosis (hydronephrosis grade = 0 on day 14 after DBJ removal) after URS. Supplementary Table 2: impact of baseline eGFR on changes in urinary renal damage markers in both groups. [file 5505969.f1.pdf]

Supplementary Fig 1. (A, B) Impact of ureteral stone size on uNGAL, u-αGST, and u-πGST levels in the ureteral stone group; **(C)** Comparing the urine biomarkers at different time periods between subgroups of stones ≦1 cm and stones >1 cm in group 2.

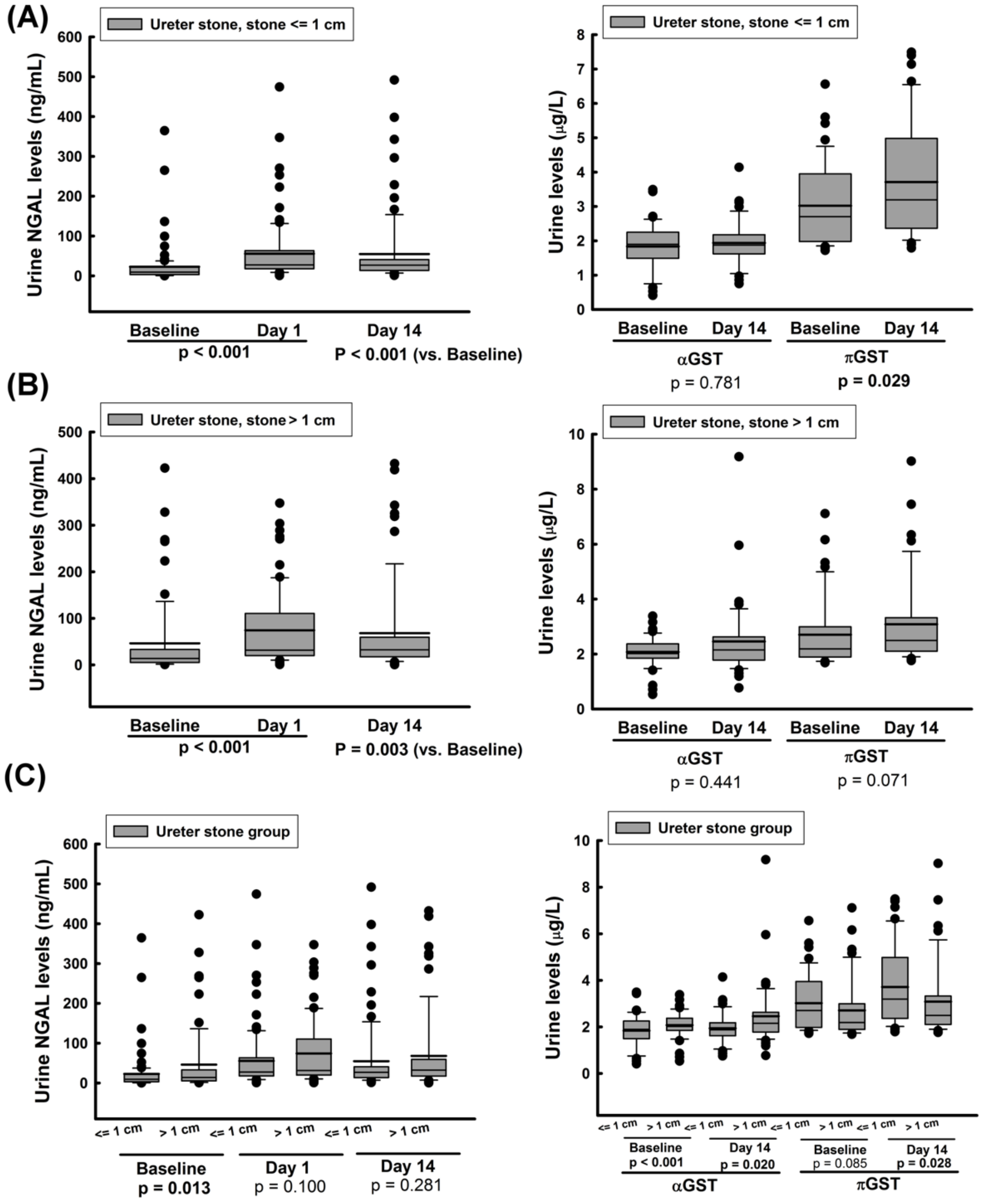

Supplementary table 1. Univariate and multivariate logistic regression analysis for the predictors of complete recovery of hydronephrosis (hydronephrosis grade =0 on Day 14 after DBJ removal) after URS.

	Univariate		Multivariate	
	OR (95% CI)	*p* value	OR (95% CI)	*p* value
Age (year)	0.97 (0.95, 0.99)	0.005*	0.96 (0.94, 0.99)	0.002*
Female (vs. Male)	0.76 (0.42, 1.36)	0.354		
Group (vs. ureteral stricture)				
Ureteral stone	2.49 (0.30, 4.77)	0.246		
Staghorn stone	2.92 (0.81, 10.5)	0.102		
Stone position				
Upper	0.62 (0.18, 2.11)	0.448		
Middle	0.70 (0.16, 2.98)	0.630		
Lower	0.71 (0.20, 2.53)	0.596		
eGFR (Baseline)	1.02 (1.01, 1.03)	0.004*	1.04 (1.02, 1.06)	<0.001*
BMI (kg/m^2)				
18.5-24 (vs. <=18.5)	2.17 (0.40, 11.8)	0.371		
24-30 (vs. <=18.5)	1.43 (0.28, 7.38)	0.670		
>30 (vs. <=18.5)	2.57 (0.42, 15.9)	0.310		
Stone size				
0.4-1.0 (vs. <0.4)	2.22 (1.06, 4.66)	0.035*	1.90 (0.81, 4.44)	0.138
>1.0 (vs. <0.4)	2.15 (1.07, 4.30)	0.031*	2.56 (1.13, 5.77)	0.024*
Presence of UTI	0.91 (0.51, 1.63)	0.742		
NGAL(Baseline)	1.00 (1.00, 1.00)	0.357		
NGAL(1-day)	1.00 (1.00, 1.00)	0.317		
NGAL(2-wk)	1.00 (1.00, 1.00)	0.209		
Δ NGAL	1.00 (1.00, 1.00)	0.234		
πGST (Baseline)	1.06 (0.77, 1.45)	0.744		
πGST (2-wk)	1.02 (0.97, 1.06)	0.521		
Δ πGST	1.00 (1.00, 1.01)	0.204		
αGST (Baseline)	0.83 (0.42, 1.66)	0.599		
αGST (2-wk)	0.97 (0.93, 1.01)	0.091		
Δ αGST	1.00 (1.00, 1.00)	0.780		

Serum Creatinine (Baseline)	1.02 (0.99, 1.04)	0.203

Abbreviations: NGAL, neutrophil gelatinase-associated lipocalin; GST, glutathione S-transferas; Δ NGAL, ΔNGAL = day 14 NGAL − baseline NGAL; 2-wk, 2 weeks after URS.

Impact of hydronephrosis degree on eGFR and urine biomarkers

Supplementary table 2. Impact of baseline eGFR on changes in urinary renal damage markers in both groups.

Baseline eGFR (mL/min/1.73 m^2)	Ureteral stricture		Ureteral stone	
	≧60 n = 40	<60 n = 11	≧60 n = 108	<60 n = 33
ΔNGAL	23.8±9.2	63.2±24.1	25.6±4.9	6.0±20.1
p value	0.132		**0.014**	
ΔαGST	0.8±0.4	0.7±0.7	0.1±0.1	0.5±0.4
p value	0.857		0.768	
ΔπGST	0.5±0.2	-0.3±0.6	0.7±0.2	0.1±0.2
p value	0.180		0.216	

p value was analyzed by *t*-test if the variables were normally distributed; the Mann-Whitney U-test was used for data that were not normally distributed., $P<0.05$ was considered statistically significant.

ΔNGAL = Day 14 NGAL − Day 0 NGAL.

Bold value represents statistically significant $p <0.05$.
